# Lipofuscin accumulation and autophagy in glaucomatous human lamina cribrosa cells

**DOI:** 10.1186/1471-2415-14-153

**Published:** 2014-12-02

**Authors:** Elizabeth M McElnea, Emily Hughes, Aloysius McGoldrick, Amanda McCann, Barry Quill, Neil Docherty, Mustapha Irnaten, Michael Farrell, Abbot F Clark, Colm J O’Brien, Deborah M Wallace

**Affiliations:** University College Dublin School of Medicine and Health Sciences, University College Dublin, Dublin, Ireland; Institute of Ophthalmology, Mater Misericordiae University Hospital, Dublin, Ireland; Conway Institute of Biomolecular and Biomedical Research, University College Dublin, Dublin, Ireland; Department of Physiology, School of Medicine, Trinity Biomedical Sciences Institute, Trinity College, Dublin, Ireland; Department of Neuropathology, Beaumont Hospital, Dublin, Ireland; Department of Cell Biology & Anatomy and the North Texas Eye Research Institute, University of North Texas, Fort Worth, Texas USA

**Keywords:** Glaucoma, Lipofuscin, Oxidative stress, Lamina cribrosa, Autophagy

## Abstract

**Background:**

Disease associated alterations in the phenotype of lamina cribrosa (LC) cells are implicated in changes occurring at the optic nerve head (ONH) in glaucoma. Lipofuscin, the formation of which is driven by reactive oxygen species (ROS), is an intralysosomal, non-degradable, auto-fluorescent macromolecule which accumulates with age and can affect autophagy - the lysosomal degradation of a cell’s constituents. We aimed to compare the content of lipofuscin-like material and markers of autophagy in LC cells from normal and glaucoma donor eyes.

**Methods:**

The number and size of peri-nuclear lysosomes were examined by transmission electron microscopy (TEM). Cellular auto-fluorescence was quantified by flow cytometry. Cathepsin K mRNA levels were assessed by PCR. Autophagy protein 5 (Atg5) mRNA and protein levels were analysed by PCR and Western blot. Protein levels of subunits of the microtubule associated proteins (MAP) 1A and 1B, light chain 3 (LC3) I and II were analysed by Western blot. Immunohistochemical staining of LC3-II in ONH sections from normal and glaucomatous donor eyes was performed.

**Results:**

A significant increase in the number of peri-nuclear lysosomes [4.1 × 10,000 per high power field (h.p.f.) ± 1.9 vs. 2.0 × 10,000 per h.p.f. ± 1.3, p = 0.002, n = 3] and whole cell auto-fluorescence (83.62 ± 45.1 v 41.01 ± 3.9, p = 0.02, n = 3) was found in glaucomatous LC cells relative to normal LC cells. Glaucomatous LC cells possessed significantly higher levels of Cathepsin K mRNA and Atg5 mRNA and protein. Enhanced levels of LC3-II were found in both LC cells and optic nerve head sections from glaucoma donors.

**Conclusions:**

Increased lipofuscin formation is characteristic of LC cells from donors with glaucoma. This finding confirms the importance of oxidative stress in glaucoma pathogenesis. Intracellular lipofuscin accumulation may have important effects on autophagy the modification of which could form the basis for future novel glaucoma treatments.

## Backround

Primary open angle glaucoma (POAG) is the commonest of a diverse group of progressive optic neuropathies which cumulatively affect approximately 67 million people worldwide [1]. The structure of the lamina cribrosa (LC) of the optic nerve head (ONH) is disrupted in response to elevated intraocular pressure (IOP) in POAG [2]. Altered LC biomechanics may interrupt axoplasmic flow in retinal ganglion cell (RGC) axons on their path through the LC (the mechanical theory of glaucoma) [3] and/or reduce the perfusion pressure in the blood vessels of this region (the vascular theory of glaucoma) [4]. In either case the end result is optic disc cupping, RGC death and, in turn, the visual field losses characteristic of glaucoma [5].

We have already demonstrated the contribution of LC cells which are found within the LC and do not express either glial fibrillary acid protein (GFAP) or other markers definitive for microglia [6], to the extracellular matrix (ECM) remodeling of the ONH in POAG [7–9]. Glaucoma related stimuli elicit expression of well described (collagens, elastin, versican, biglycan, vascular endothelial growth factor) and novel (LOX, emmprin, thrombomodulin) ECM genes in these cells [7, 8, 10].

POAG is predominantly a disease of aging [11]. Throughout the body age related changes in cell structures are thought to result from damage caused by reactive oxygen species (ROS) [12]. Mitochondria are the most important endogenous sources of ROS. Oxidative phosphorylation in these organelles results in electron leak. This provides for the continuous formation of ROS and, in turn, the direct exposure of the mitochondrion to these ROS [13, 14]. When cellular ROS production overwhelms cellular antioxidant defenses, oxidative stress ensues. The presence of oxidative stress is a common feature of many neurodegenerative diseases. The means by which oxidative stress may induce RGC death in glaucoma is acknowledged to include not only the direct neurotoxic effects of ROS but also the indirect damage by oxidative stress of glial cells [11]. We have previously shown that the mitochondria of LC cells derived from donors with glaucoma have reduced mitochondrial membrane potential in keeping with mitochondrial dysfunction and that increased ROS production is a feature of these cells [15].

Autophagy refers to lysosomal degradation of a cell’s own constituents [16]. A double membraned structure containing engulfed cytoplasm and its organelle content - the autophagosome, fuses with lysosome(s) to create an autophagolysosome within which the endocytosed contents can be degraded by lysosomal enzymes [17, 18]. Non-functional mitochondria accumulate with age. Cell homeostasis is maintained by removing damaged mitochondria in the autophagic process of mitophagy [19]. Even under the most favourable of conditions however, the turnover of compromised organelles is incomplete so that, with age, there is a gradual intralysosomal accumulation of waste material. The oxidative modification of this material results in the formation of a complex, non degradable, electron dense, auto-fluorescent substance called lipofuscin [20–22]. Lipofuscin formation is thus ROS driven. Oxidatively stressed trabecular meshwork (TM) cells and retinal pigment epithelium (RPE) cells challenged with ROS have been shown to accumulate lipofuscin [23, 24].

Lipofuscin cannot be degraded by or extruded from cells [25, 26]. Advanced cellular lipofuscin accumulation diminishes intracellular lysosomal degradative capacity by preventing lysosomal enzymes from targeting functional autophagolysosomes. Newly produced lysosomal enzymes are directed to lipofuscin loaded lysosomes in a futile attempt to degrade their un-degradable material [27]. This eventually further limits mitochondrial recycling. The compromised lysosomal system is unable to effectively remove either oxidatively damaged structures or the defective mitochondria whose ROS production promotes such damage [16].

The presence of lipofuscin in the optic nerve has previously been described [28]. More recently, the almost exclusive localization of this compound to within the cytoplasm of the glial cells located within the axonal fascicles of the nerve has been confirmed [29]. Optic nerves from patients with POAG were found to have significantly higher concentrations of lipofuscin than those derived from healthy donors and it was postulated that the relatively higher concentration of this compound in glaucomatous optic nerves might exacerbate the optic neuropathy [29].

This study aimed to compare the levels of lipofuscin-like material and markers of autophagy in LC cells from normal eyes (NLC) with those from eyes with glaucoma (GLC). We found that cellular lipofuscin content is increased in LC cells from eyes with glaucoma. In doing so we confirm the importance of oxidative stress and mitochondrial dysfunction in glaucoma pathogenesis that has already been highlighted by our group [15]. Autophagy likely also plays an important role in preventing disease related changes occurring at the ONH in glaucoma. Here we show that cellular markers of autophagy are increased in LC cells from eyes with glaucoma relative to those from normal donors.

## Methods

### LC cell culture

Eyes were obtained from the Lion’s Eye Institute for Transplant and Research (LEITR), Tampa, Florida, United States of America (USA), donors or a first degree relative having given consent for their use for research purposes and LC cell explants from the same subsequently donated by Alcon Laboratories, Fort Worth, Texas, USA. The acquisition of these cells was carried out in accordance with the declaration of Helsinki.

Isolation of LC cells was carried out on eyes received within 24 hours of death. The cell lines used were characterized by immune fluorescence staining for a number of glial cell markers. Cells were deemed to be LC cells if they stained positively for elastin, fibronectin, laminin, collagen I, collagen III and collagen IV, α-smooth muscle actin and if they stained negatively for GFAP. Further, cells were tested for GFAP positivity by RT-PCR as per Lambert *et al.* using a primer set that amplifies a 285 base pair region of GFAP cDNA and were found to be negative [30].

LC cell lines from a total of five different donors with no history of glaucoma and ages ranging from 68 to 91 years, mean age 81.0 ± 10.2 years and from a total of four different donors with glaucoma and ages ranging from 68 to 83 years, mean age 77.8 ± 6.4 years were used in this study. For each experimental procedure, cell lines from each of three different donors with no history of glaucoma and from each of three donors with a history of glaucoma were used unless otherwise indicated.

Cells were cultured as described by Hernandez et al. [6]. Briefly, cultures were maintained in Dulbecco’s modified eagle medium (DMEM) supplemented with 10% (v/v) fetal calf serum, 200 mM L-glutamine, 10,000 units/ml penicillin and 10 mg/ml streptomycin (Sigma, Ireland). Cultures were used in experimental procedures between passages 4 and 8. For each experiment only cell lines within one passage of one another were used.

### Transmission electron microscopy

Cells were trypsinized and washed twice in PBS before fixing in 2.5% glutaraldehyde in 0.1 M sodium cacodylate buffer (pH 7.2) for 4 hours at 4°C. The fixative was subsequently decanted and replaced with 0.1 M sodium cacodylate buffer and the sample re-suspended and left for 2 hours. The buffer was then decanted and replaced with a solution of 1% agarose in distilled water and the samples re-suspended before centrifugation at 10,000 rotations per minute (r.p.m.) for 1 minute. Cell samples were detached, post-fixed in 1% osmium tetroxide in 0.1 M sodium cacodylate buffer and processed for transmission electron microscopy (TEM) in the Neuropathology Department at Beaumont Hospital. 65 nm sections were examined. The quantity of lipofuscin-like lysosomes in images of 10 randomly selected peri-nuclear fields from different cells at magnifications of 7450X and 22300X were recorded for each cell line. The area of each of the lipofuscin-like lysosomes identified was calculated using ImageJ software.

### Measurement of endogenous cellular auto-fluorescence

Endogenous cellular auto-fluorescence was detected under the FITC filter by fluorescence microscopy. The fluorescence emitted by approximately 10,000 cells in the FL-1 channel (563–607 nm wavelength band) was quantified by flow cytometry (Beckman Coulter Cyan ADP) and analyzed using Summit 4.3 software.

### RNA extraction and cDNA preparation

Total RNA was extracted using Tri-Reagent (Invitrogen, Ireland) extraction, chloroform phase separation and isopropanol precipitation. Complimentary deoxyribonucleic acid (cDNA) was generated by reverse transcription of 0.5 μg of DNAase treated total ribonucleic acid (RNA) using the random primer method (Invitrogen). Products were visualized on 1% ethidium bromide stained agarose gels.

### Cathepsin K and ATG5 RT-PCR

Gene specific exon-exon spanning primers for Cathepsin K and ATG5 were designed by qPrimerDepot as follows, Cathepsin K forward 5′ – CATTTAGCTGCCTTGCCTGT – 3′ and reverse 5′ – TACATGACCAATGCCTTCCA – 3′ and Atg5 forward 5′ – ACTGTCCATCTGCAGCCAC – 3′ and reverse 5′ -GCCATCAATCGGAAACTCAT – 3′. PCR was carried out on a PerkinElmer 7700 cycler using the following steps 1) 10 minutes at 95°C; 2) 10 seconds at 95°C; 3) 20 seconds at 55°C; 4) 20 seconds at 72°C; and 5) repeat from 3) for an additional 39 times. The absence of nonspecific products was confirmed by analysis of the melt curves and by electrophoresis in 1% agarose gel as described below. Gene expression rates were compared using 18S rRNA normalized threshold cycle number values (cT’s). In the case of Cathepsin K, NLC samples were assigned an arbitrary value of 1. The equation 2-^ΔcT^ was used to derive a fold difference for levels of Cathepsin K mRNA in GLC compared to NLC.

### Agarose gel electrophoresis

PCR samples were visualized by electrophoresis in 1% agarose gel. Products were visualized by staining with ethidium bromide (0.5 mg/ml) in the agarose and subsequent illumination on a 302 nm UV trans-illuminator. The molecular size standard (100 base pair ladder) was included.

### Western blot analysis

Total protein from LC cells was obtained by lysing in RIPA buffer (50 mM Tris–HCl pH 7.4, 1% NP40, 0.25% sodium deoxycholate, 150 mM NaCl, 1 mM EGTA, 1 mM sodium orthovanadate, 1 mM sodium fluoride) containing antipain (1 μg/ml), aprotinin (1 μg/ml), chymostatin (1 μg/ml), leupeptin (0.1 μg/ml), pepstatin (1 μg/ml) and PMSF (0.1 mM). The total amount of protein in each sample was determined using the Bio-Rad protein assay (Bio-Rad, United Kingdom) with bovine serum albumin as standard. Between 30-40 μg of total protein was electrophoresed on 15% polyacrylamide gels followed by transfer to nitrocellulose membrane (Schleicher and Schuell, Germany) and incubated overnight at 4°C with the appropriate antibodies (Cell Signaling Technology LC3A/B Antibody #4108 and Atg5 Antibody #2630,United Kingdom) including β-actin (Cell Signaling Technology #4967) as a loading control. Bands were detected by incubation with a rabbit secondary antibody conjugated to horseradish peroxidase (Cell Signaling #7074) and development carried out by enhanced chemiluminescence (ECL) (Amersham, United Kingdom).

### Immunohistochemistry

Human optic nerve head sections were again donated by Alcon Laboratories, Fort Worth, Texas, USA, having been initially obtained from the Lion’s Eye Institute for Transplant and Research. ONH sections from three different donors with no history of glaucoma and from three different donors with glaucoma were used in this study. Again, informed consent for the use of such tissue was given and its acquisition carried out in accordance with the declaration of Helsinki. As before, ethical approval for their use as described here was received from the University of North Texas Health Science Centre.

Staining for the detection of LC3-II was carried out on 5 μm thick longitudinal sections of human ONH which were air dried onto glass slides and paraffin embedded as has been described previously [31]. Briefly, sections were deparaffinised in xylene and descending alcohol series. Epitope retrieval was performed using 10 mM Tris-EDTA pH 9.0 for 4 minutes in a pressure cooker at full pressure mode. Endogenous peroxidase activity was blocked using BLOXALL endogenous peroxidase and alkaline phosphatase blocking solution (#SP-6000, Vector Laboratories, USA). Sections were then exposed 1: 100 to primary mouse monoclonal LC3 antibody (#5 F10, Nanotools antibodies, Germany) for 45 minutes at room temperature, before rinsing with Tris-buffered Tween. Secondary antibody treatment with Dako Envision kit (# K400, Dako Diagnostics, Dublin, Ireland) followed for 30 minutes. This reaction was terminated using 3, 3′-diaminobenzidine tetrahydrochloride for 10 minutes. Specimens were counterstained with Haem Z haematoxylin (RBA-4201-00A, Cellpath, United Kingdom) for 5 minutes, and dipped in acid alcohol (1%) followed by Scott’s tap water substitute. Finally, sections were dehydrated through ascending alcohol series before cleaning with xylene and mounting.

### Statistical analysis

Data are represented as the mean ± SD and were analyzed using the Student’s *t*-test. P ≤ 0.05 was considered statistically significant and is indicated in the appropriate figures by _*_.

## Results

### Lipofuscin accumulates in LC cells from donors with POAG

**Figure 1:**
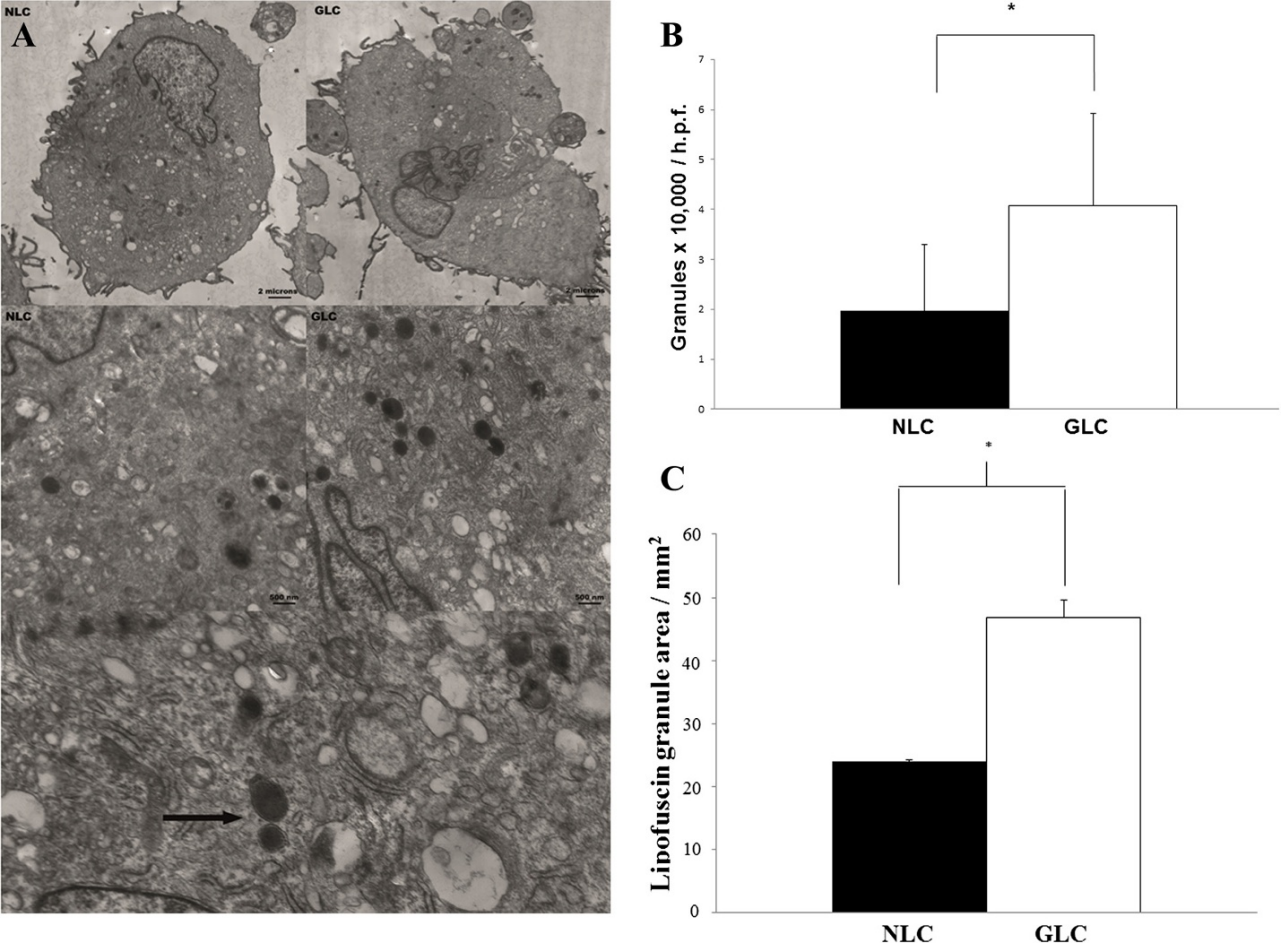
**The ultra-structural appearance of LC cells and their accumulation of lipofuscin. A**: The TEM images shown demonstrate the ultra-structural appearance of normal and glaucomatous LC cells at low (7450X) and high (22300X) power magnifications. Note the abundance, particularly in LC cells from the glaucoma donors, of predominantly peri -nuclear, lysosome-like, membrane bound organelles containing an amorphous, electron dense material suggestive of lipofuscin. Two such structures have been indicated in the lowest frame by an arrow. **B**: Quantification of lipofuscin-like lysosomes in each of 10 randomly selected peri-nuclear fields at magnifications of 7450X and/or 22300X were recorded for normal and glaucomatous LC cells. These structures were more numerous in LC cells from glaucoma donors compared to those from normal donors (4.1 × 10,000 per h.p.f. ± 1.9 vs. 2.0 × 10,000 per h.p.f. ± 1.3, p = 0.002, n = 3). **C**: The area of each of the lipofuscin like lysosomes identified for the construction of Figure 1B as described above was calculated twice using ImageJ software and a mean value for each recorded. Lipofuscin occupied a larger cell area in glaucomatous LC samples when compared to normal LC samples (46.8 mm^2^ ± 2.9 vs. 23.9 mm^2^ ± 0.3, p = 0.07, n = 3).

Transmission electron microscopy identified the intracellular peri-nuclear accumulation of membrane bound organelles containing amorphous, electron dense material - phenotypic features suggestive of lipofuscin granules, at magnifications of both 7450X and 22300X in both normal and glaucomatous LC cells. Representative electron micrographs are shown in Figure 1A. These structures were more numerous in LC cells from glaucoma donors (4.1 × 10,000 per h.p.f. ± 1.9 vs. 2.0 × 10,000 per h.p.f. ± 1.3, p = 0.002, n = 3) as shown in Figure 1B. The total area occupied by these bodies was greater in glaucomatous LC cells (46.8 mm^2^ ± 2.9 vs. 23.9 mm^2^ ± 0.3, p = 0.007, n = 3) as can be seen from Figure 1C.

**Figure 2:**
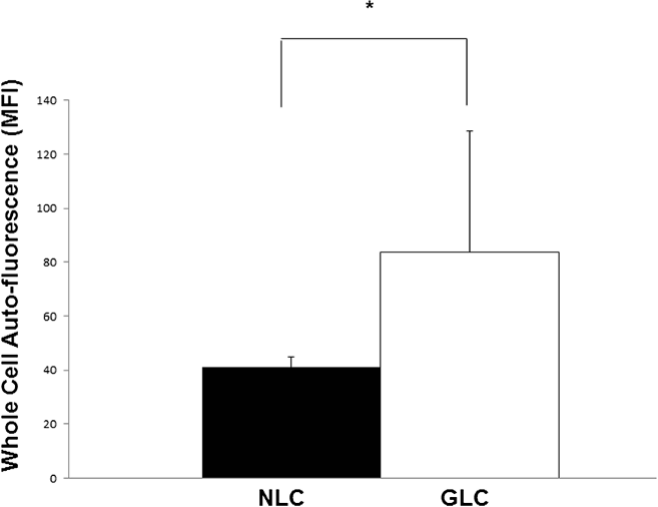
**LC cell auto-fluorescence reflecting cellular lipopfuscin content.** The lipofuscin auto-fluorescence emitted by approximately 10,000 cells in the yellow-green range of the spectrum (563-607 nm, FL-1 channel) was quantified by flow cytometry. Glaucomatous LC cells demonstrated increased whole cell auto-fluorescence relative to normal LC cells (83.6 ± 45.1 MFI vs. 41.0 ± 3.9 MFI, p = 0.02, n = 3).

Intracellular auto-fluorescent material was found under the FITC filter at live cell fluorescence microscopy in LC cells from both normal and glaucoma donors. As shown in Figure 2, whole cell auto-fluorescence, reflective of cellular lipofuscin content [32] and measured at the 563-607 nm wavelength band, was increased in GLC [auto-fluorescence 83.6 ± 45.1 mean fluorescence intensity (MFI)] compared to NLC (auto-fluorescence 41.0 ± 3.9 MFI, p = 0.02, n = 3) cell groups. The data from Figures 1B, C and 2 has been summarized in Table 1.

**Table 1:** **This table summarizes the data from Figures** 1 **B, C and** 2 **and shows how the number and area of the lipofuscin-like granules and the auto-fluorescence thought secondary to the same was greater in lamina cribrosa cells derived from donors with glaucoma compared to lamina cribrosa cells from donors with no history of glaucoma**

	NLC ***Mean ± SD***	GLC ***Mean ± SD***	p-value ≤
Number of lipofuscin-like granules (× 10,000 per h.p.f)	2.0 ± 1.3	4.1 ± 1.9	0.002
Area of lipofuscin-like granules (mm^2^)	23.9 ± 0.3	46.8 ± 2.9	0.007
Autofluorescene (M.F.I.)	41.0 ± 3.9	83.62 ± 45.1	0.02

GLC = Glaucomatous lamina cribrosa, M.F.I Mean flourescene intensity, NLC = Normal lamina cribrosa, SD = Standard deviation.

### Lysosomal function in lipofuscin loaded LC cells from donors with POAG

**Figure 3:**
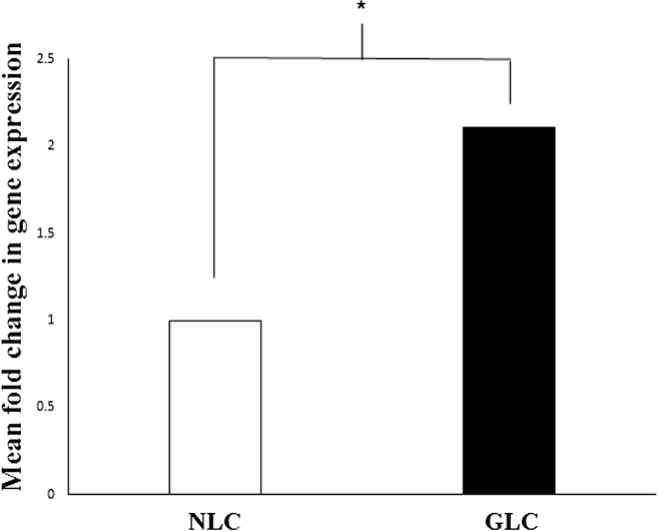
**mRNA levels of the lysosomal cysteine protease Cathepsin K were quantified by real-time PCR analysis and normalized with 18 s RNA expression.** There was a significant increase in Cathepsin K mRNA expression for three LC samples from glaucoma donors relative to three samples from donors without glaucoma with a mean fold difference of 2.1 ± 1.2, p = 0.04.

Expression rates for the CTSK gene encoding for Cathepsin K were compared using 18S rRNA normalized threshold cycle number values (cT’s). The average cT of the replicates from normal donors was assigned a value of one. Subtracting the NLC mean calibrator cT from each individual glaucoma donor sample yields ΔcT. The equation 2-^ΔcT^ was used to derive a fold difference for levels of Cathepsin K RNA in glaucomatous LC cells compared to normal LC cells. As is demonstrated in Figure 3, we found greater expression of the CTSK gene at RT-PCR analysis in LC cells from glaucoma donors relative to samples from normal donors with a mean fold change of 2.1 ± 1.2, p = 0.04, n = 3.

### Autophagy in lipofuscin loaded LC cells from donors with POAG

**Figure 4:**
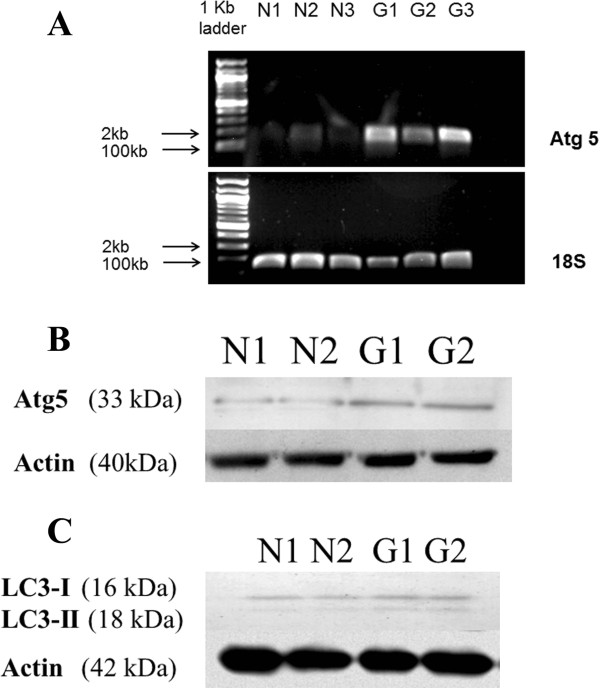
**Enhanced cellular autophagy in glaucomatous LC cells. A**: Levels of ATG5 mRNA were quantified by real-time PCR analysis. Gene expression rates were compared using 18S rRNA normalized threshold cycle number values (cT’s). PCR samples were visualized by electrophoresis in 1% agarose gel. As can be seen from the agarose gel shown, elevated levels of ATG5 mRNA were found in glaucomatous LC cells (n = 3) relative to NLC (n = 3). **B**: Elevated levels of Atg5 protein in glaucoma LC cells relative to normal LC cells were confirmed by Western blotting. Actin was included as a loading control. **C**: While both normal LC (n = 2) and glaucomatous LC cells (n = 2) had approximately equal quantities of LC3-I protein, in the Western blot given we observe an increase in LC3-II in LC cells from glaucoma donors.

The level of Atg5 in a cell is used as a marker for that cell’s autophagic activity. We found increased levels of Atg5 mRNA in glaucomatous LC cells. The levels of Atg5 in normal LC cells were undetectable and so we were unable to calculate threshold cycle number values (cT’s) for all normal samples and so, in turn could not provide a fold difference for the quantity of Atg5 mRNA in glaucomatous LC cells compared to normal LC cells. To overcome this we visualized the Atg5 PCR samples following electrophoresis in a 1% ethidium bromide stained agarose gel as is shown in Figure 4A. Our findings of increased levels of Atg5 mRNA in LC cells from glaucoma donors translated into elevated Atg5 protein as can be seen in Figure 4B.

**Figure 5:**
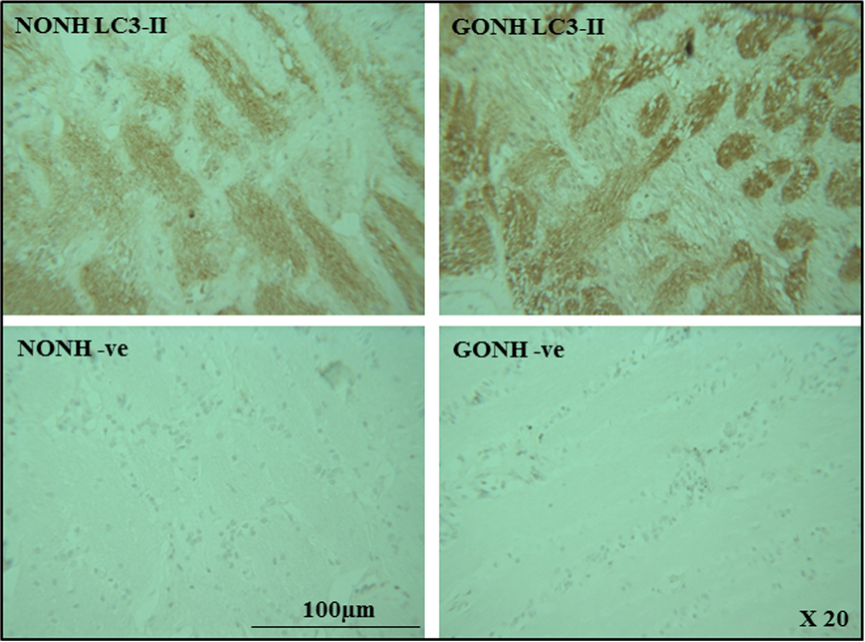
**Immunohistochemical staining of ONH sections with the autophagy marker LC3 II.** Longitudinal sections through optic nerves from glaucomatous (GONH) and normal (NONH) donors at magnifications of 20X stained for LC3-II are shown. Cell nuclei stain purple-blue. Positive staining appears brown and is more abundant in that section from the donor with glaucoma.

Light chain 3 (LC3) is a subunit of microtubule associated proteins 1A and 1B (MAP1LC3) [33]. Cleavage of LC3 at its carboxy terminus immediately following its synthesis yields the cytosolic LC3-I form [34, 35]. During autophagy, LC3-I is converted to LC3-II through lipidation by an ubiquitin-like system involving autophagy proteins 3 (Atg3) and 7 (Atg7). This allows LC3 to become localized to autophagic vesicles [36, 37]. Thus the conversion of LC3-I to the lower migrating LC3-II is used to monitor autophagy [35]. Figure 4C shows that we found elevated levels of LC3-II protein in glaucomatous LC cells.Further, as Figure 5 illustrates, immnuohistochemical staining of ONH sections for LC3-II was more pronounced in sections from donors with a history of glaucoma (GONH) compared to sections taken from donors with no history of glaucoma (NONH).

## Discussion

Lipofuscin is primarily composed of protein and lipid that has accumulated within lysosomes. It is thought to be found in some quantity in all cells [38–40]. The oxidative modification of this material renders it un-degradable. As can be seen in Figure 1A, when observed by TEM, the most obvious feature of the GLC cells of this study was the increased presence of cytoplasmic, membrane bound organelles containing an electron dense material. Altogether, their peri-nuclear location, spherical lysosome-like morphology and the luminal presence of electron dense material make these structures consistent with lipofuscin, a terminal product of the cellular lysosomal pathway. These organelles were more numerous and larger in size than those found in NLC cell cultures. Lipofuscin is auto-fluorescent. As Figure 2 shows GLC demonstrated increased cellular auto-fluorescence, which again is reflective of their increased lipofuscin content.

An accumulation of lipofuscin loaded lysosomes has been demonstrated in porcine TM cells exposed to hyperoxic conditions (40% 0_2_) [32]. That the intracellular accumulation of lipofuscin in the GLC samples of our study occurred in a cellular system that we have already demonstrated to have a depleted complement of antioxidants [15] demonstrates the potential for such a phenomenon *in vivo*.

The accumulation of un-degradable lipofuscin in autophagic lysosomes has been proposed to result in increased lysosomal mass with consequent increases in cellular lysosomal enzyme content [41]. Porcine TM cells have been shown to respond to the accumulation of lipofuscin with the synthesis of novel lysosomes and lysosomal enzymes [32]. When these TM cells were placed under conditions of chronic oxidative stress, an increase in cellular levels of Cathepsin K mRNA and protein was found. In our study, as can be seen from Figure 3, we also demonstrate a significant difference in levels of the lysosomal enzyme Cathepsin K mRNA in GLC compared to NLC.

During autophagy, autophagosomes engulf cytoplasmic components. A cytosolic form of LC3-I is conjugated >to phosphatidylethanolamine to form LC3-II which is recruited to the autophagosomal membrane [35]. Autophagosomes fuse with lysosomes to form autophagolysosomes and intra-autophagolysosomal components are degraded by lysosomal enzymes. At the same time, LC3-II in the autophagolysosomal membrane is degraded. Turnover of LC3-II thus reflects autophagic activity and detecting LC3 by Western blot has become a method of monitoring this process [42]. In humans, the complex of Autophagy protein 5 (Atg5) with autophagy proteins 12 (Atg12) and 16 (Atg16), is also necessary for the formation of the autophagosome. Consequently, cellular levels of Atg5 are indicative of autophagic activity [43, 44]. In this study, as Figure 4 shows, we found increased levels of Atg5 mRNA and protein and elevated levels of LC3-II protein in our glaucomatous LC cells. While their increased expression does not guarantee that the whole autophagic process i.e. the fusion of autophagosomes with lysosomes is functioning appropriately, it may be that GLC cells are responding to their accumulation of lipofuscin with the synthesis of new autophagolysosmes in an attempt to ensure appropriate turnover of defunct organelles and with that, cellular homeostasis.

Defects in autophagy have already been detected in many age-related pathologies including a growing number of neurodegenerative diseases. Axotomy of the optic nerve is a classic model of neurodegeneration [45]. Muela *et al.* found that autophagy was activated shortly after optic nerve axotomy in mice. As here, they demonstrated up-regulation of Atg5 and increases in LC3-II and concluded that enhanced autophagy is likely highly relevant in milder situations of axonal damage such as glaucoma. They proffered the modulation of autophagy as a promising future clinical target in the amelioration of neurodegenerative disorders [46].

## Conclusion

In conclusion then, the accumulation of lipofuscin is a feature of LC cells from eyes with glaucoma. This suggests again that alterations in mitochondrial function and oxidative stress are important in the development of POAG. The accumulation of lipofuscin may alter cellular autophagic activity. Elevated levels of cellular markers of autophagy are found in glaucomatous LC cells. Future anti-glaucoma strategies may aim to reduce oxidative stress and/or improve mitochondrial turnover through the stimulation of cellular degradation systems at the level of the LC.

## Abbreviations

Atg3: Autophagy protein 3
Atg5: Autophagy protein 5
Atg7: Autophagy protein 7
Atg16: Autophagy protein 16
cT’s: Threshold cycle number
DMEM: Dulbecco’s modified eagle medium
ECL: Enhanced chemiluminesence
ECM: Extracellular matrix
GFAP: Glial fibrillary acid protein
GLC: Glaucomatous lamina cribrosa
GONH: Glaucomatous optic nerve head
IOP: Intraocular pressure
LC: Lamina cribrosa
LC3: Light chain 3
LEITR: Lion’s eye institute for transplant and research
MAP1: Microtubule associated protein 1
NLC: Normal lamina cribrosa
NONH: Normal optic nerve head
ONH: Optic nerve head
POAG: Primary open angle glaucoma
RGC: Retinal ganglion cell
ROS: Reactive oxygen species
RPE: Retinal pigment epithelium
TEM: Transmission electron microscopy
TM: Trabecular meshwork.
